# Conduction System Pacing Versus Biventricular Pacing for Cardiac Resynchronization Therapy: A Systematic Review and Meta‐Analysis of Randomized Controlled Trials

**DOI:** 10.1002/joa3.70500

**Published:** 2026-10-21

**Authors:** Shreyas Singireddy, Sami Shoura, Darshine Venugopal, Ragavendar Saravanabavanandan, Kyle Coombes, Timir Baman, Siddharth Shah

**Affiliations:** ^1^ Division of Cardiology, Department of Internal Medicine University of Illinois College of Medicine at Peoria Peoria Illinois USA

**Keywords:** biventricular pacing, cardiac resynchronization therapy, conduction system pacing, left bundle branch pacing, meta‐analysis

## Abstract

**Background:**

Conduction system pacing (CSP) is an emerging alternative to biventricular pacing (BVP) for cardiac resynchronization therapy (CRT). Recent randomized controlled trials (RCTs) have yielded divergent results, creating uncertainty about the comparative effectiveness.

**Methods:**

We performed a systematic review and meta‐analysis of RCTs comparing CSP with BVP for CRT, searching PubMed, Embase, and CENTRAL through May 2026 (updating a prior review). The primary outcome was change in left ventricular ejection fraction (ΔLVEF), analyzed with random‐effects models and prediction intervals.

**Results:**

Eight parallel‐group RCTs including 799 patients (396 CSP, 403 BVP) were included. Pooled ΔLVEF did not differ significantly (MD +2.40%, 95% CI −0.49 to 5.29, *p* = 0.091; *I* ^2^ = 75%). CSP showed a non‐significant trend toward shorter paced QRS (MD −7.6 ms, *p* = 0.14) and improved NYHA class (SMD −0.33, *p* = 0.12). Heart failure hospitalization (RR 0.69), mortality (RR 1.17), complications (RR 0.84), and procedure time did not differ. The four trials enrolling exclusively LBBB patients favored CSP (MD +5.00%, 95% CI 3.01 to 6.98, *p* = 0.004; *I* ^2^ = 0%), consistent in the three using confirmed LBBAP/LBBP (MD +5.37%, 95% CI 3.23 to 7.52, *p* = 0.008; *I* ^2^ = 0%).

**Conclusions:**

In this analysis of randomized trials of CRT, no statistically significant difference was observed between CSP and BVP for any outcome. Because no non‐inferiority margin was prespecified and the clinical‐event analyses were underpowered, these findings should not be interpreted as establishing equivalence or comparable safety. Exploratory analyses suggest that patients with LBBB undergoing LBBAP/LBBP may derive incremental benefit, warranting confirmation in adequately powered trials.

## Graphical abstract

Current randomized evidence does not demonstrate statistically significant superiority of CSP over BVP for CRT. Exploratory analyses suggest benefit may be greatest among patients with true LBBB receiving dedicated LBBAP/LBBP.

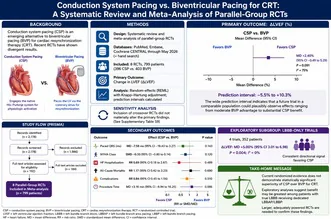

## 1 Introduction

Cardiac resynchronization therapy (CRT) with biventricular pacing (BVP) is a guideline‐recommended treatment for patients with heart failure, reduced ejection fraction, and conduction delay [1, 2]. Despite robust evidence supporting BVP, approximately 30% of patients do not respond, in part due to suboptimal lead positioning and non‐physiologic activation [3].

Conduction system pacing (CSP)—including His bundle pacing (HBP), left bundle branch area pacing (LBBAP), and left bundle branch pacing (LBBP)—offers an alternative strategy that engages the intrinsic His‐Purkinje network [4, 5]. Over the past several years, a growing number of RCTs have compared CSP with BVP for CRT, with variable results. Some trials demonstrate signals of CSP benefit, particularly in left bundle branch block (LBBB) populations [6, 7, 8], while the PhysioSync‐HF trial reported CSP inferiority in a multicenter Brazilian cohort using mixed CSP strategies [9].

This divergence raises questions about whether CSP and BVP produce similar resynchronization on average, whether certain CSP subtypes or patient populations may derive differential benefit, and whether procedural and definitional heterogeneity explains the inconsistency. We therefore performed a systematic review and meta‐analysis of all published parallel‐group RCTs comparing CSP with BVP for CRT.

## 2 Methods

### 2.1 Search Strategy and Study Selection

This systematic review was conducted per PRISMA 2020 guidelines (checklist, Table S6) and registered with PROSPERO on 27 May 2026 (CRD420261407189). The review was conducted between 30 April and 30 June 2026. The analysis plan was amended during the review (recorded in the PROSPERO version history) from the originally registered DerSimonian–Laird model (with Mantel–Haenszel risk ratios for binary outcomes) to restricted maximum likelihood (REML) with the Knapp–Hartung adjustment across all outcomes, for more reliable inference with few studies; DerSimonian–Laird, Mantel–Haenszel, and Peto analyses are reported as sensitivity analyses. The registered primary outcome was change in LVEF; paced QRS duration and the remaining measures were analyzed as secondary outcomes. Additional sensitivity analyses added after registration are labeled post hoc/exploratory (Table S1). This review updates the prior systematic review of Felix et al. [10]. PubMed, Embase, and Cochrane CENTRAL were searched from July 2023 (the end of the Felix et al. search) through May 15, 2026 (full strategy, including complete Embase syntax, in Table S1). Randomized trials identified by Felix et al. were carried forward and re‐adjudicated against our eligibility criteria; two crossover‐design trials were moved to sensitivity analyses and the LEVEL‐AT cohort was replaced by its extended‐follow‐up report (CONSYST‐CRT). ClinicalTrials.gov and reference lists were hand‐searched, with no language restrictions. The full search strategy for each database is provided in Table S1.

We included parallel‐group RCTs comparing any form of CSP with BVP in adults with a CRT indication. Crossover‐design RCTs were excluded from the primary analysis but retained for sensitivity. When extended follow‐up existed for the same cohort (CONSYST‐CRT 12‐month for LEVEL‐AT 6‐month [11, 12]), only the most recent publication was included.

### 2.2 Data Extraction and Risk of Bias

Two reviewers (SS, S. Sh.) independently extracted study‐level data (per‐study values in Tables S3 and S4). SDs were imputed from 95% CIs where necessary. Outcomes were extracted on an intention‐to‐treat basis wherever reported. For His‐SYNC (Upadhyay [13]), which had 48% crossover, the arm sizes analyzed (16 CSP, 24 BVP) reflect the as‐treated groups reported in the primary publication rather than the original randomized allocation; a sensitivity analysis excluding high‐crossover trials (≥ 25%: His‐SYNC, CONSYST‐CRT) is reported. CSP subtype was classified as His bundle pacing (HBP); left bundle branch area pacing (LBBAP; capture of the left bundle branch area, potentially including left ventricular septal capture without electrocardiographically confirmed left bundle capture); left bundle branch pacing (LBBP; with confirmed left bundle branch capture per consensus criteria); hybrid conduction‐system CRT (e.g., His‐optimized CRT [HOT‐CRT], in which a coronary‐sinus/left ventricular lead supplements conduction‐system pacing); or mixed strategies. Trial‐level definitions and capture‐confirmation status are detailed in Table S9. Risk of bias was assessed using Cochrane RoB 2 across five domains (Table S5, Figures S2 and S3).

### 2.3 Outcomes

The primary outcome was ΔLVEF (%). The ΔLVEF at each trial's primary assessment time point was used (6 months in most trials; 12 months in CONSYST‐CRT and PhysioSync‐HF, which reported at that time point). Where multiple time points were available (HeartSync‐LBBP [8]: 6 and 36 months), the 6‐month value was used; a longest‐available‐follow‐up sensitivity analysis (substituting HeartSync‐LBBP 36‐month data) yielded a pooled MD of +2.38% (95% CI −0.51 to 5.28), essentially unchanged from the primary estimate (Table S8). Secondary outcomes included paced QRS (ms), NYHA class (SMD), heart failure hospitalization, all‐cause mortality, complications, procedure time, and pacing threshold.

### 2.4 Statistical Analysis

Random‐effects meta‐analysis used restricted maximum likelihood (REML) estimation with the Knapp–Hartung adjustment, which provides more appropriate confidence interval coverage than DerSimonian–Laird when the number of studies is small [14]. Mean difference (MD) was used for continuous outcomes on the same scale; Hedges' *g* for NYHA; risk ratios (RR) for binary outcomes with 0.5 continuity correction for zero cells. Studies with zero events in both arms were excluded from binary outcome analyses. Given sparse events, we additionally performed sensitivity analyses for binary outcomes using the Peto odds ratio and the Mantel–Haenszel risk difference (Table S8). 95% prediction intervals were calculated for all outcomes [15]. DerSimonian–Laird was performed as sensitivity for comparison with prior literature.

Heterogeneity was quantified using *I* ^2^ and *τ* ^2^. Given that only eight trials were available, neither funnel plots nor formal tests of publication bias (e.g., Egger's test) were undertaken, as both are underpowered and unreliable when the number of studies is small (*k* < 10) [16]. Exploratory sensitivity analyses included: (a) exclusion of PhysioSync‐HF; (b) restriction to trials enrolling exclusively (100%) LBBB patients, and within these to trials using confirmed LBBAP/LBBP; (c) restriction to LBBAP/LBBP trials; (d) restriction to standalone conduction system pacing, defined by exclusion of hybrid (HOT‐CRT) and mixed/deep‐septal strategies (i.e., HBP, LBBAP, and LBBP trials), capture confirmation not being an inclusion criterion for this analysis and being tabulated separately in Table S9; and (e) exclusion of high‐crossover trials (≥ 25%). These analyses are exploratory and hypothesis‐generating; primary inference derives from the overall pooled estimate. Meta‐regression examined LBBB proportion and CSP subtype as potential moderators, acknowledging limited power (*k* = 8). Leave‐one‐out analysis and influence diagnostics were performed. The certainty of evidence for each major outcome was rated using the GRADE framework (Table S11). All analyses used R (v4.5) with the “meta” (v8.3) and “metafor” (v4.8) packages.

## 3 Results

### 3.1 Study Selection and Characteristics

Eight parallel‐group RCTs enrolling 799 patients (396 CSP, 403 BVP) met inclusion criteria (Figure S1; Table 1; detailed characteristics in Table S2). Two crossover trials were identified but excluded from primary analysis [20, 21]. Follow‐up ranged from 6 to 36 months. CSP subtypes included HBP (2 trials: His‐SYNC, His‐Alternative), LBBAP (3: [6], CONSYST‐CRT, CSP‐SYNC), strict LBBP (1: HeartSync‐LBBP), hybrid HOT‐CRT (1: [18]), and mixed CSP (1: PhysioSync‐HF). Four trials enrolled exclusively LBBB patients (Table S9).

**TABLE 1:** Characteristics of included trials.

Study	*N*	CSP type	FU (mo)	Sites	Country	LBBB %	NICM %	Age (y)	Female %	BL LVEF%	BL QRS ms
Upadhyay [13]	40	HBP	12	4	USA	82	NR	65	37	27	170
Vinther [17, 18]	50	HBP	6	1	Denmark	100	78	66	36	29	172
Wang [6]	40	LBBAP	6	1	China	100	100	64	50	29	172
Vijayaraman [19]	100	HOT‐CRT	6	1	USA	71	61	70	31	28	155
Pujol‐López [11]	134	LBBAP	12	1	Spain	56	66	69	27	28	175
Žižek [7]	62	LBBAP	6	1	Slovenia	100	68	68	30	29	174
Chen [8]	200	LBBP	36	6	China	100	82	65	32	28	168
Zimerman [9]	173	Mixed^a^	12	14	Brazil	95	87	62	50	26	180

Abbreviations: BL, baseline; FU, follow‐up; NICM, non‐ischemic cardiomyopathy; NR, not reported.

^a^ Mixed: 69% LBBAP, 20% deep septal, 9% LOT‐CRT, 2.5% His.

Risk of bias was low in three of eight trials [6, 7, 8]. Five trials had “some concerns,” primarily related to crossover (up to 48%) or unblinded outcome assessment. No trial was rated high risk (Figures S2, S3, Table S5).

### 3.2 Primary Outcome: Change in LVEF

All eight trials reported ΔLVEF. The pooled estimate showed a non‐significant trend favoring CSP (MD +2.40%, 95% CI −0.49 to 5.29, *p* = 0.091; *I* ^2^ = 75%; *τ* ^2^ = 9.28; prediction interval −5.5 to + 10.3%) (Figure 1). The wide prediction interval indicates that a future trial in a comparable population could plausibly observe effects ranging from moderate BVP advantage to substantial CSP benefit.

**FIGURE 1:**
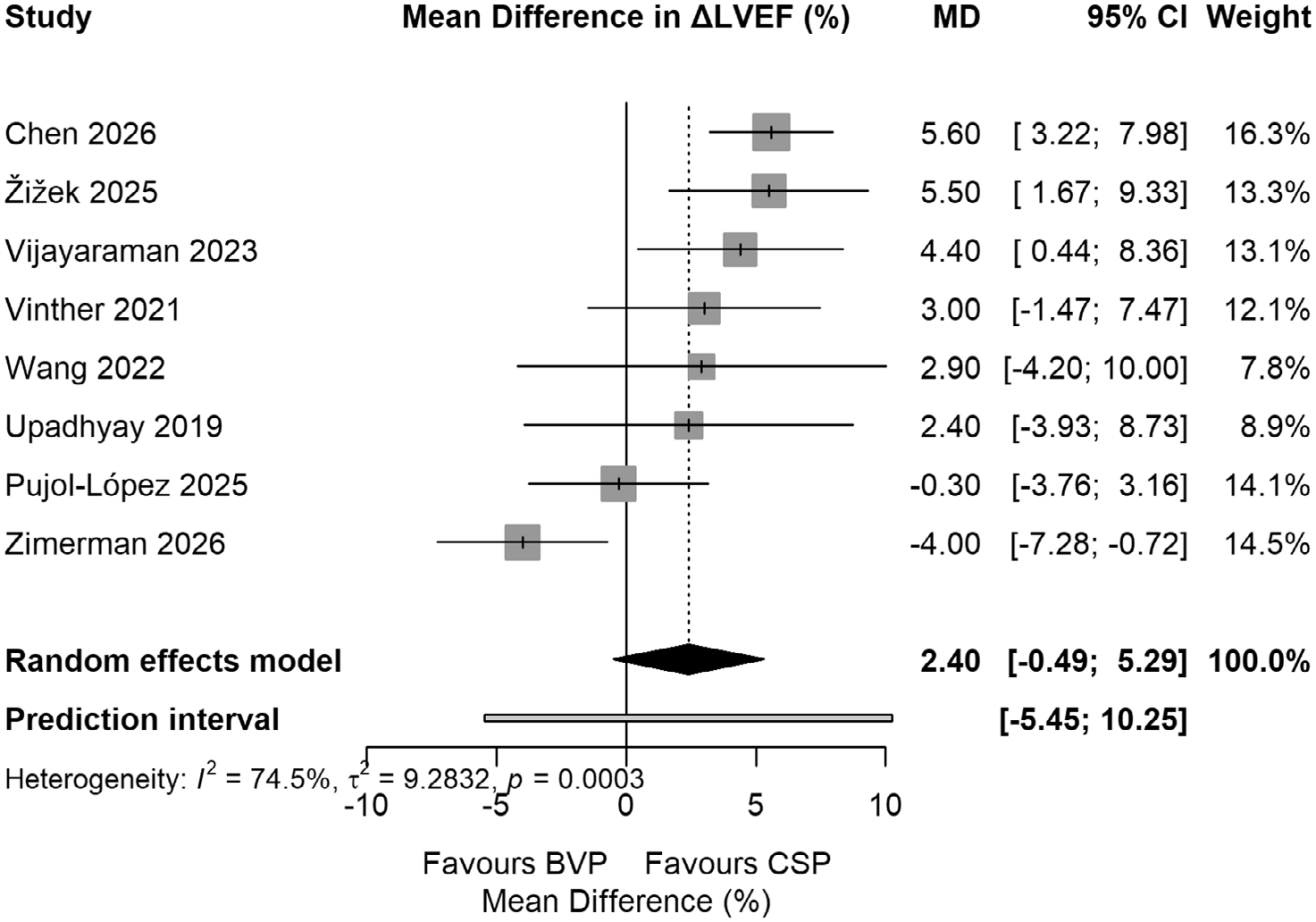
Forest plot of ΔLVEF (%). Positive MD favors CSP. Gray band = 95% prediction interval. REML + Knapp–Hartung.

Leave‐one‐out analysis identified Zimerman 2026 (PhysioSync‐HF) as the most influential study: its exclusion changed the pooled MD to +3.61% (95% CI 1.49 to 5.73, *p* = 0.006) and reduced *I* ^2^ from 75% to 32% (Table S7). Influence diagnostics flagged this study as an outlier (studentized residual −3.48). No other study exclusion changed significance.

**TABLE 2:** Summary of pooled results (REML + Knapp–Hartung).

Outcome	*k*	*N*	Measure	Effect (95% CI)	*p*	*I* ^2^%	PI
ΔLVEF (%)	8	799	MD	2.40 (−0.49, 5.29)	0.091	75	−5.5, 10.3
Paced QRS (ms)	8	799	MD	−7.58 (−18.43, 3.27)	0.143	85	−36, 21
NYHA class	5	597	SMD	−0.33 (−0.80, 0.14)	0.120	67	−1.2, 0.6
Procedure time	8	799	MD	3.16 (−9.94, 16.25)	0.586	60	−24, 30
Threshold (V)	8	799	MD	−0.22 (−0.56, 0.12)	0.170	90	−0.9, 0.5
HF hosp.	5^a^	669	RR	0.69 (0.18, 2.67)	0.489	69	0.04, 11.1
Mortality	5^a^	669	RR	1.17 (0.42, 3.23)	0.690	11	—
Complications	8	799	RR	0.84 (0.45, 1.54)	0.510	0	—

*Note:* PI = 95% prediction interval. *N* = 669 denotes the contributing trials. Prediction intervals are omitted for all‐cause mortality and complications, for which between‐study heterogeneity was minimal (*I* ^2^ ≤ 11%) and the resulting intervals uninformative given the small number of events.

Abbreviations: MD, mean difference; RR, risk ratio; SMD, standardized mean difference (Hedges' *g*).

^a^ For heart failure hospitalization and all‐cause mortality, *k* = 5 reflects exclusion of two trials with zero events in both arms (His‐Alternative [6]) and of His‐SYNC (events not separately reported).

### 3.3 Secondary Outcomes

#### 3.3.1 Paced QRS Duration

CSP showed a non‐significant trend toward shorter paced QRS (*k* = 8, MD −7.58 ms, 95% CI −18.43 to 3.27, *p* = 0.14; *I* ^2^ = 85%) (Figure 2). While the point estimate favors CSP, the Knapp–Hartung correction appropriately widens confidence intervals given the small number of heterogeneous studies; using the DerSimonian–Laird estimator, this difference reached nominal significance (MD −7.36 ms, 95% CI −14.11 to −0.61, *p* = 0.033).

**FIGURE 2:**
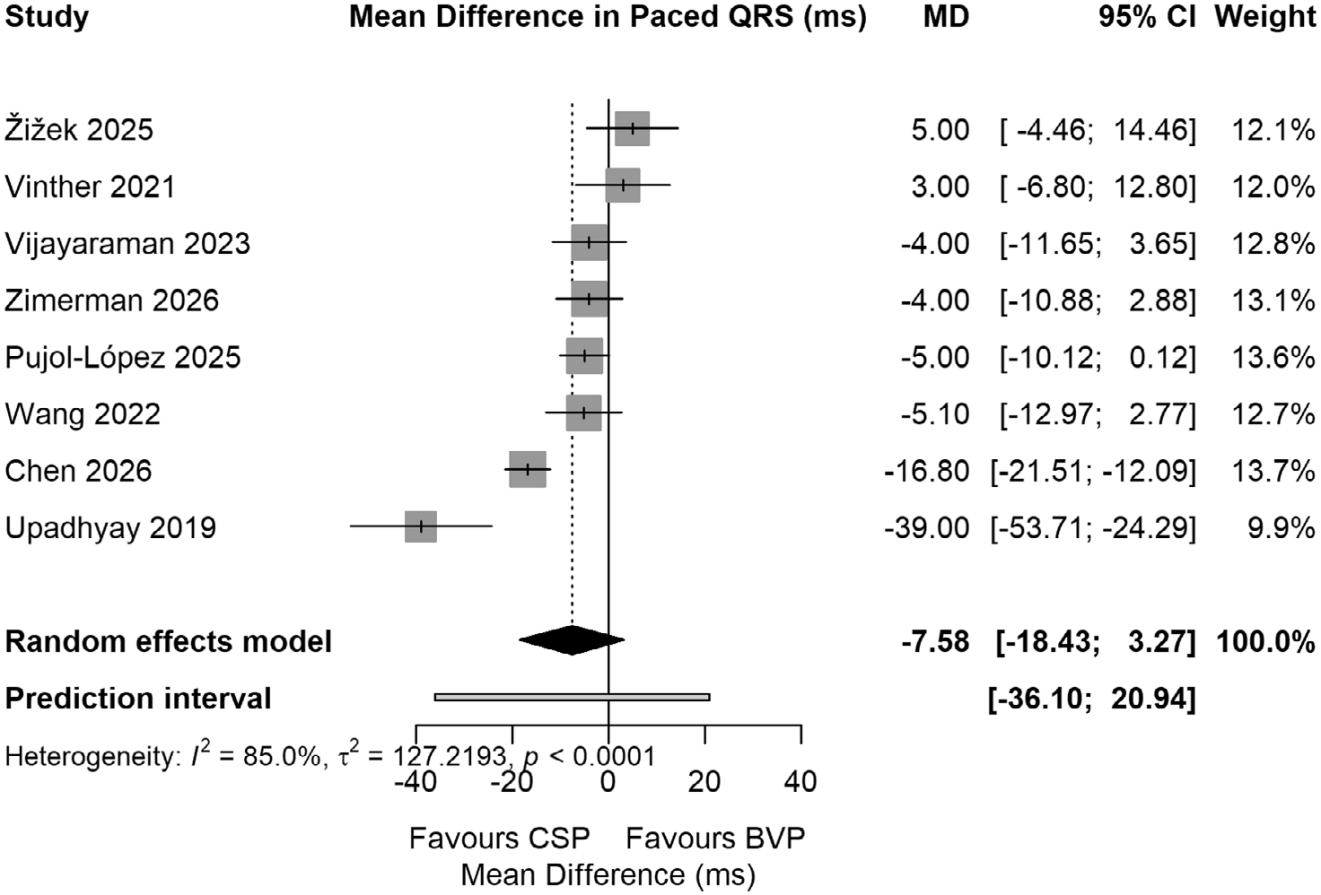
Forest plot of paced QRS (ms). Negative MD = shorter QRS with CSP.

#### 3.3.2 NYHA Functional Class

Among five reporting trials, CSP showed a non‐significant trend toward greater functional improvement (SMD −0.33, 95% CI −0.80 to 0.14, *p* = 0.12; *I* ^2^ = 67%) (Figure 3).

**FIGURE 3:**
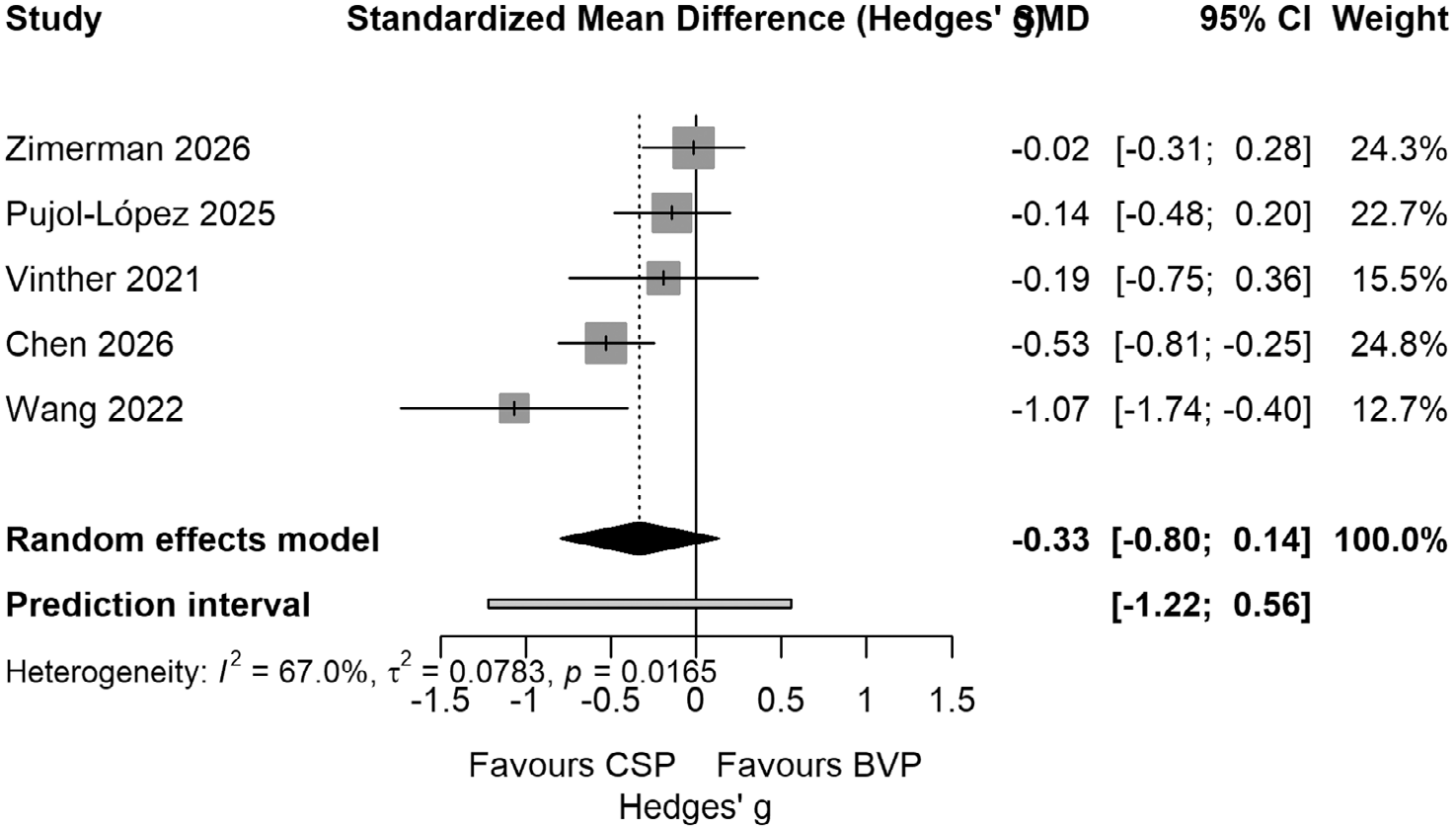
Forest plot of NYHA class (Hedges' *g*). Negative SMD = improvement with CSP.

#### 3.3.3 Clinical Events

Five trials each contributed to the heart failure hospitalization and mortality analyses (studies with zero events in both arms excluded). Heart failure hospitalization (RR 0.69, 95% CI 0.18 to 2.67, *p* = 0.49; *I* ^2^ = 69%), all‐cause mortality (RR 1.17, 95% CI 0.42 to 3.23, *p* = 0.69; *I* ^2^ = 11%), and complications (*k* = 8, RR 0.84, 95% CI 0.45 to 1.54, *p* = 0.51; *I* ^2^ = 0%) did not differ significantly (Figures 4, 5, 6). Across all trials, 36 deaths and 76 HFH events occurred, underscoring the limited power for these endpoints. In sparse‐data sensitivity analyses the Mantel–Haenszel risk differences were small and non‐significant for all binary outcomes; the Peto odds ratio for HFH was 0.51 (95% CI 0.32 to 0.83), but the Peto method is biased when arm sizes and event counts are imbalanced (as here, driven by HeartSync‐LBBP), so it does not alter the underpowered, non‐significant primary result (Table S8).

**FIGURE 4:**
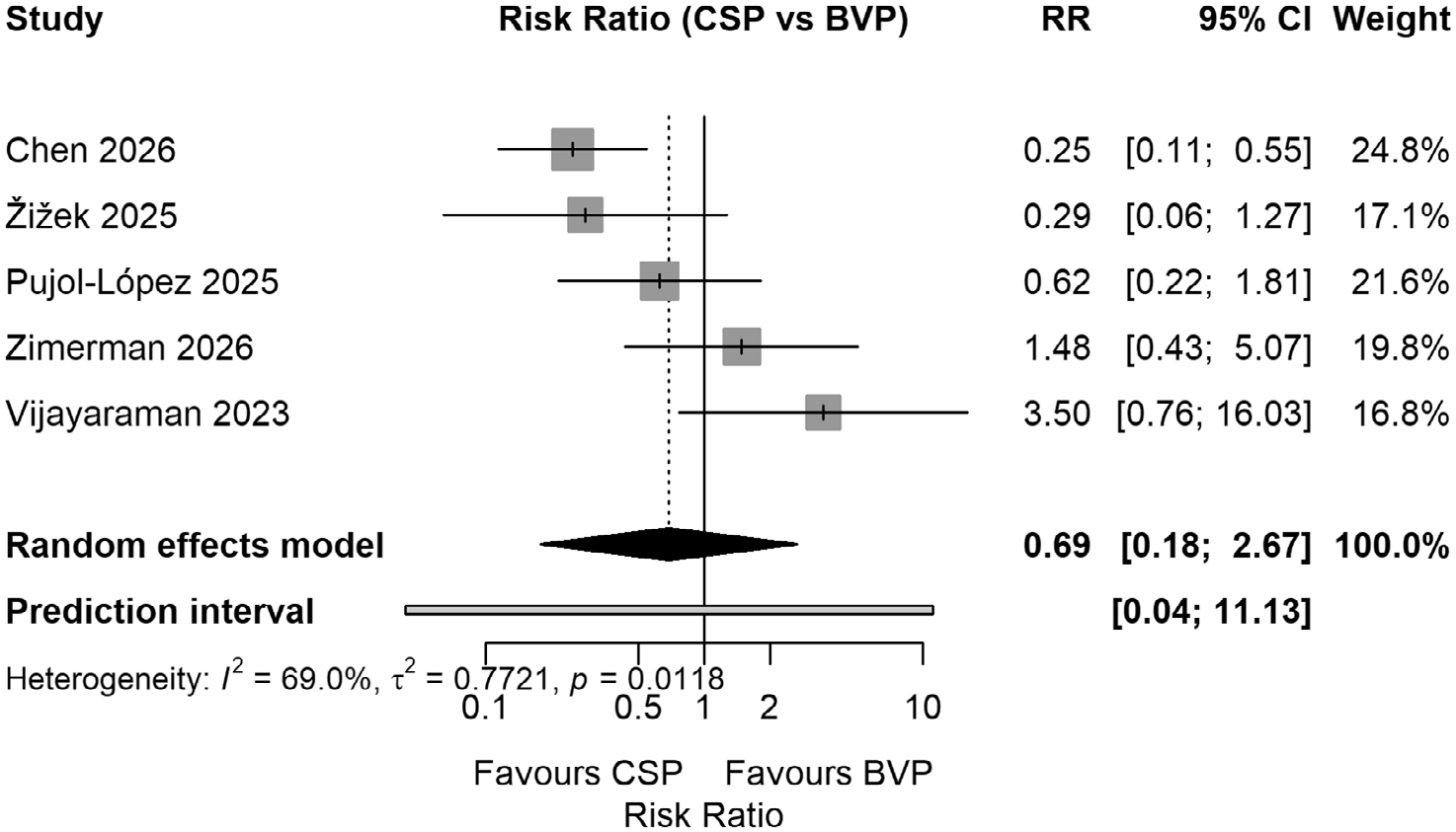
Forest plot of HF hospitalization (RR).

**FIGURE 5:**
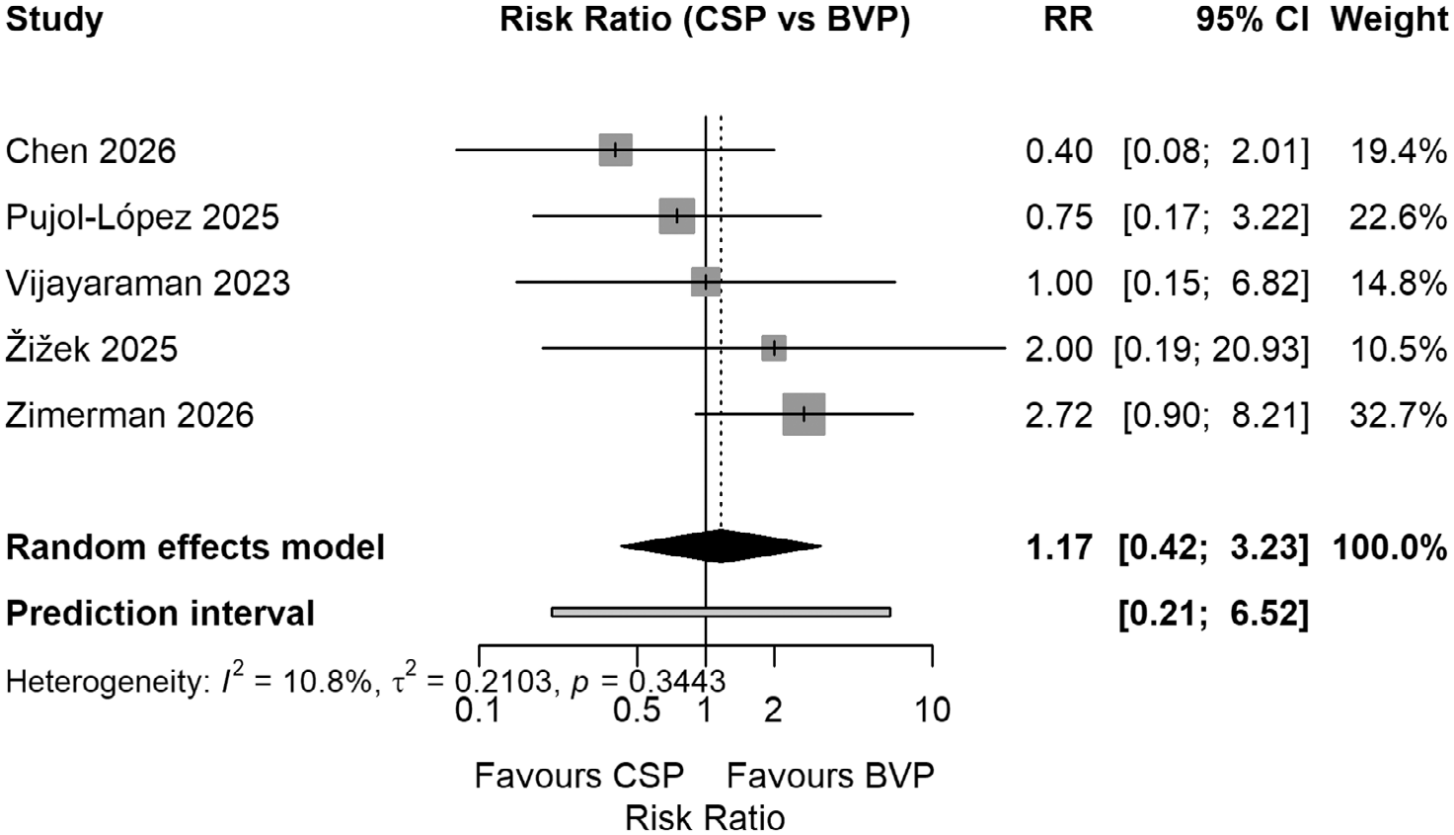
Forest plot of all‐cause mortality (RR).

**FIGURE 6:**
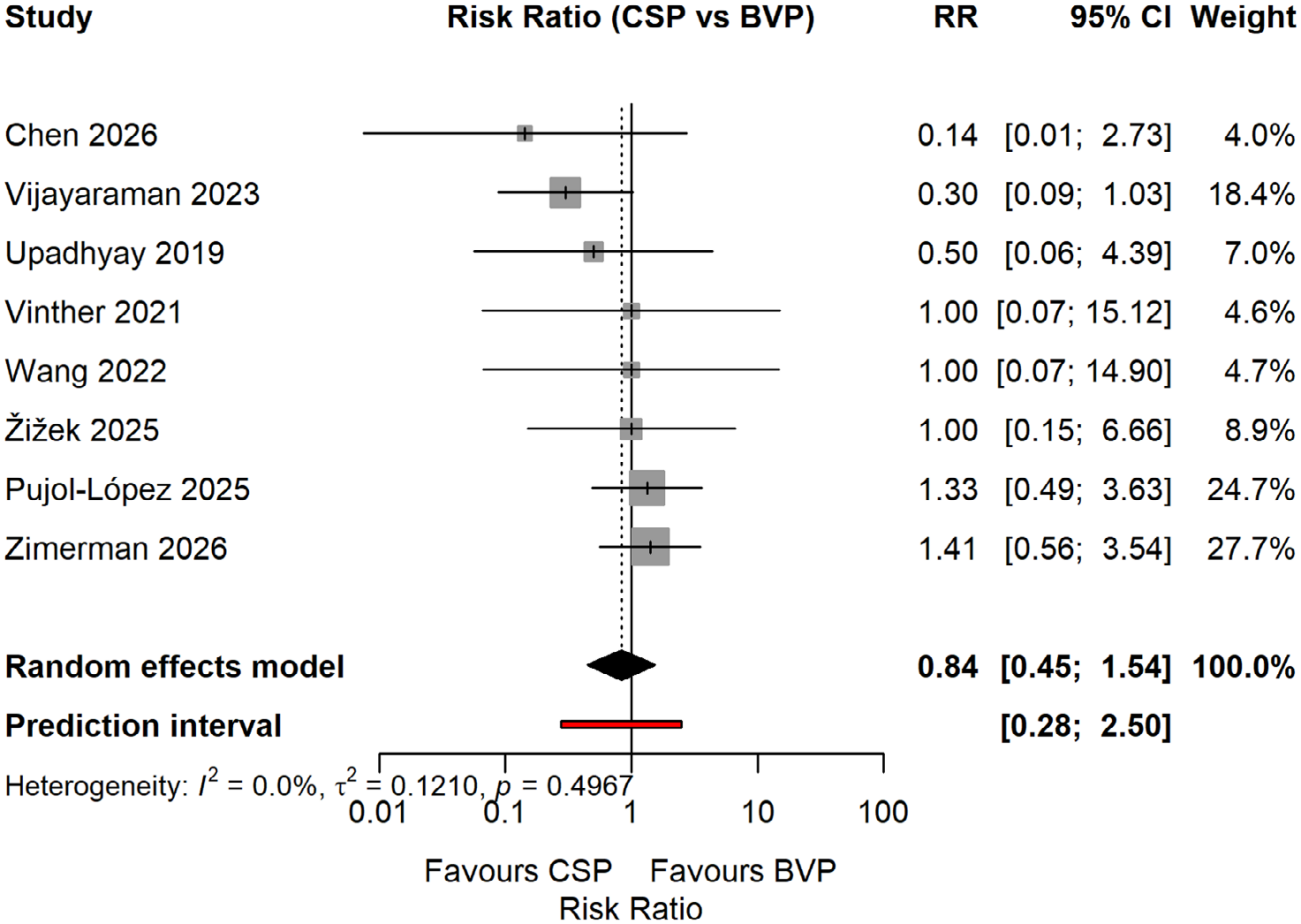
Forest plot of complications (RR).

#### 3.3.4 Procedural Outcomes

Procedure time did not differ (MD +3.2 min, 95% CI −9.9 to 16.3, *p* = 0.59; *I* ^2^ = 60%). Pacing threshold showed a non‐significant trend favoring CSP (MD −0.22 V, 95% CI −0.56 to 0.12, *p* = 0.17; *I* ^2^ = 90%) (Table 2; Figure 7).

**FIGURE 7:**
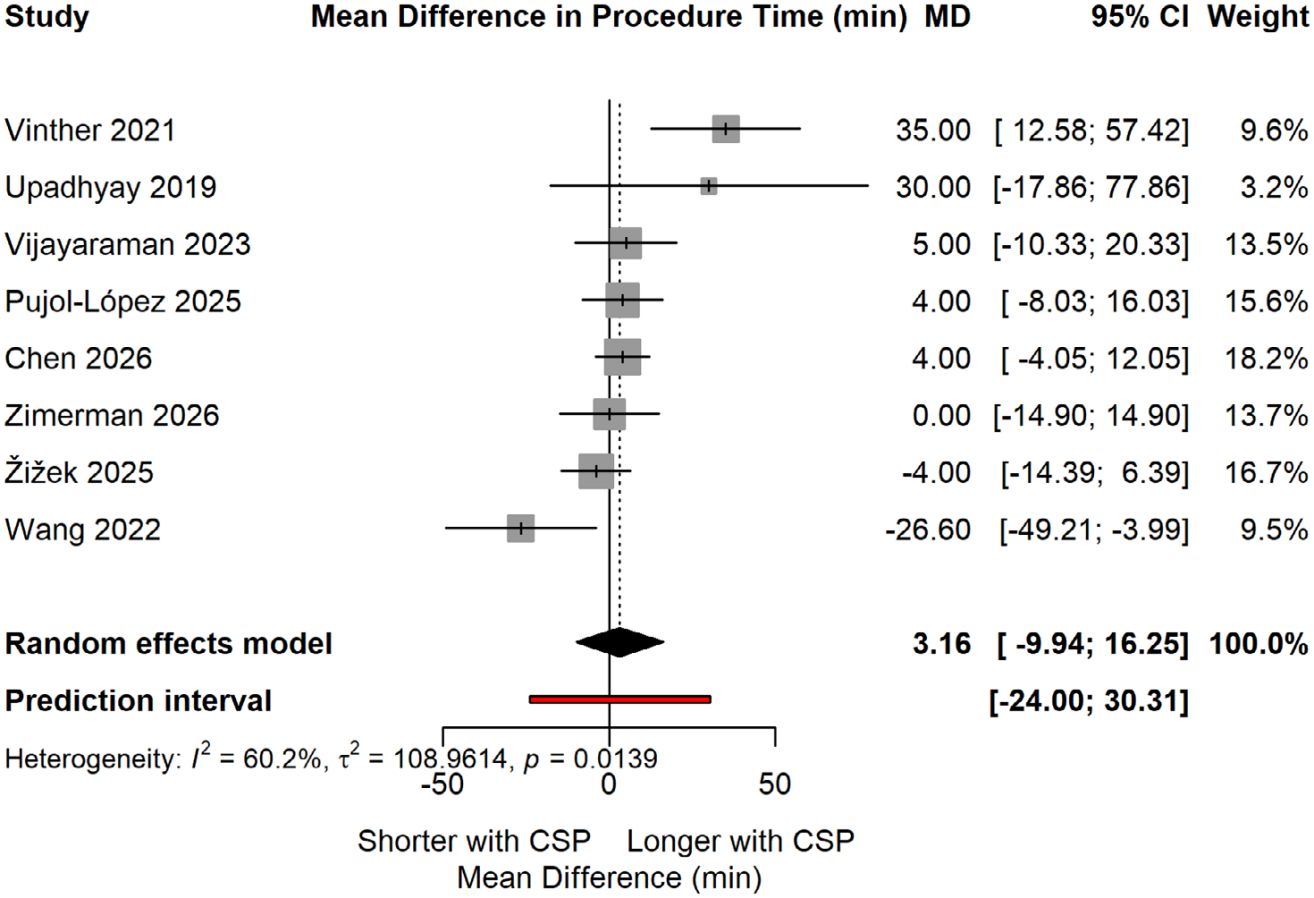
Forest plot of procedure time (min).

### 3.4 Exploratory Sensitivity Analyses

Excluding PhysioSync‐HF (*k* = 7, *N* = 626) yielded MD +3.61% (95% CI 1.49 to 5.73, *p* = 0.006; *I* ^2^ = 32%). Restricting to the four trials enrolling exclusively LBBB patients yielded MD +5.00% (95% CI 3.01 to 6.98, *p* = 0.004; *I* ^2^ = 0%; *N* = 352); within these, the three trials using confirmed LBBAP/LBBP yielded MD +5.37% (95% CI 3.23 to 7.52, *p* = 0.008; *I* ^2^ = 0%; *N* = 302). Restricting to standalone conduction system pacing, excluding hybrid HOT‐CRT and mixed/deep‐septal strategies, without requiring electrocardiographic capture confirmation (which varied across these six trials; Table S9), yielded MD +3.43% (95% CI 0.83 to 6.02, *p* = 0.019; *I* ^2^ = 43%; *k* = 6). LBBAP/LBBP trials (*k* = 4, *N* = 436) yielded MD +3.59% (95% CI −1.11 to 8.28, *p* = 0.093; *I* ^2^ = 64%). Excluding high‐crossover trials (≥ 25%: His‐SYNC, CONSYST‐CRT) attenuated the estimate to MD +2.89% (95% CI −1.07 to 6.85, *p* = 0.12; *k* = 6), consistent with crossover diluting between‐group differences (Table S8). These are exploratory findings; the primary estimate from the full dataset remains the basis for inference.

Exploratory meta‐regression of ΔLVEF on LBBB proportion showed a positive but non‐significant association (*β* = 0.05 per 1% increase, *p* = 0.53). CSP subtype as a categorical moderator did not reach significance (*p* = 0.17) and, with several subtypes represented by a single trial, was statistically unstable (*R* ^2^≈51%), consistent with definitional heterogeneity contributing to the observed *I* ^2^; with only eight trials this is hypothesis‐generating and sensitive to how hybrid strategies are classified. Using the DerSimonian–Laird estimator as sensitivity, the pooled ΔLVEF was unchanged (MD +2.40%, 95% CI −0.36 to 5.16, *p* = 0.088), and paced QRS reached nominal significance (MD −7.36 ms, *p* = 0.033).

## 4 Discussion

This meta‐analysis of eight parallel‐group RCTs enrolling 799 patients finds that CSP does not demonstrate statistically significant superiority over BVP for any outcome in CRT. Point estimates for ΔLVEF (+2.40%), paced QRS (−7.6 ms), and NYHA class (SMD −0.33) consistently favor CSP, but confidence intervals cross the null for all three, and no difference was observed for heart failure hospitalization, mortality, or complications. Yet the heterogeneity itself is informative—it maps onto a gradient of CSP definition, patient selection, and operator experience that separates trials showing clear benefit from one showing harm, and understanding this gradient may matter more than the pooled estimate alone. A structured comparison with prior meta‐analyses is provided in Table S10, and the certainty of evidence for each outcome (GRADE) in Table S11; certainty was low to very low for all outcomes, driven by heterogeneity, imprecision, and sparse events.

### 4.1 The Role of Heterogeneity

The *I* ^2^ of 75% for ΔLVEF is clinically informative rather than merely statistical noise. CSP currently encompasses HBP, LBBAP (with or without confirmed LBB capture), deep septal pacing, and LOT‐CRT—strategies with distinct electrophysiologic mechanisms. The field lacks a standardized taxonomy, and pragmatic trial definitions differ from mechanistic definitions [4, 5]. PhysioSync‐HF classified 20% of its CSP arm as deep septal pacing without confirmed LBB engagement, while HeartSync‐LBBP required strict electrophysiologic confirmation [8, 9]. Whether deep septal pacing constitutes “true” CSP is an unresolved question that directly affects how pooled data should be interpreted.

The LBBB‐only subgroup (MD +5.00%, *I* ^2^ = 0%; and MD +5.37% in the three trials with confirmed LBBAP/LBBP) is consistent with the established principle that CRT benefit concentrates in true LBBB [1]. However, this derives from only four trials (352 patients) and must be interpreted as hypothesis‐generating. Similarly, excluding PhysioSync‐HF restores significance and reduces heterogeneity, but this does not justify its exclusion from the primary estimate—it is a methodologically valid multicenter RCT.

### 4.2 Context of PhysioSync‐HF

PhysioSync‐HF warrants specific consideration as the only trial reporting CSP inferiority. Its CSP arm was the most heterogeneous (69% LBBAP, 20% deep septal, 9% LOT‐CRT); it used exclusively stylet‐driven leads, enrolled from 14 sites with variable CSP experience, and recorded three procedure‐related deaths [9]. Importantly, its BVP arm performed exceptionally well (ΔLVEF +12%), suggesting a high‐quality comparator. These factors suggest the results may reflect a specific clinical context rather than a universal CSP‐BVP comparison, but they do not diminish the trial's validity.

### 4.3 Methodological Considerations

The choice of statistical estimator has a meaningful impact on these results. Using the DerSimonian–Laird method (as in prior meta‐analyses [10]), paced QRS reaches nominal significance (*p* = 0.033). With the Knapp–Hartung adjustment—now recommended for small numbers of studies [14]—this difference is attenuated (*p* = 0.14). This discrepancy underscores the sensitivity of inference to analytic choices when *k* is small and heterogeneity is high, and argues for caution in interpreting any single pooled *p*‐value.

### 4.4 Implications

These data do not support a conclusion of CSP superiority over BVP for CRT. However, the consistent directional signal in sensitivity analyses, particularly in LBBB populations receiving confirmed conduction system engagement, identifies a plausible biological hypothesis that warrants prospective testing. Several ongoing trials (Left vs. Left, NCT05650658; His‐Alternative II) are designed to address this gap. For clinical practice, CSP remains a reasonable alternative consistent with HRS/APHRS/LAHRS guidance [4], but generalizing results from expert single‐center trials to broader practice settings requires caution. Consistent with this, a recent two‐center propensity‐weighted cohort of de novo CRT recipients found that LBBAP achieved resynchronization and clinical outcomes comparable to BVP with shorter procedure times, supporting real‐world feasibility while reinforcing the need for randomized confirmation [22].

### 4.5 Limitations

The included trials are heterogeneous in CSP subtype, follow‐up, and operator expertise. Event rates are low (36 deaths, 76 HFH across 799 patients), precluding conclusions about hard outcomes. Study‐level data limits patient‐level subgroup analyses. SDs were imputed for some trials. Crossover rates varied (2%–48%), potentially diluting effects. Meta‐regression was underpowered (*k* = 8). No trial was blinded for pacing modality. The exclusion of zero‐event studies from binary outcome analyses reduced the effective sample size for HFH and mortality (*k* = 5). Because no non‐inferiority margin was prespecified, our findings indicate absence of a detected difference rather than demonstrated equivalence.

## 5 Conclusions

In this meta‐analysis of eight parallel‐group RCTs enrolling 799 patients, no outcome demonstrates a statistically significant difference between CSP and BVP for CRT when analyzed with recommended small‐sample corrections because equivalence was not formally tested, this should not be read as demonstrating comparability. Point estimates consistently favor CSP for echocardiographic and functional outcomes, but confidence intervals are wide. Substantial heterogeneity reflects variation in CSP definition, patient selection, and operator experience. Exploratory analyses suggest that dedicated LBBAP/LBBP in LBBB populations may offer benefit—a hypothesis requiring prospective validation in adequately powered trials with clinical endpoints.

## Funding

The authors have nothing to report.

## Disclosure

The authors have nothing to report.

## Ethics Statement

This systematic review and meta‐analysis of published, de‐identified aggregate data did not require institutional review board approval.

## Conflicts of Interest

The authors declare no conflicts of interest.

## Supporting information


**Table S1:** Search strategy.
**Table S2:** Detailed study characteristics.
**Table S3:** Data extraction—continuous outcomes.
**Table S4:** Data extraction—binary outcomes.
**Table S5:** Risk of bias assessment (RoB 2)—justification.
**Table S6:** PRISMA 2020 checklist.
**Table S7:** Leave‐one‐out sensitivity analysis for ΔLVEF.
**Table S8:** Exploratory sensitivity and subgroup analyses.
**Table S9:** CSP definition and capture‐confirmation by trial.
**Table S10:** Comparison with prior meta‐analyses.
**Table S11:** GRADE certainty of evidence.
**Figure S1:** PRISMA 2020 flow diagram.
**Figure S2:** Risk of bias traffic light plot.
**Figure S3:** Risk of bias summary bar chart.
**Figure S4:** Forest plot of pacing threshold (V).

## Data Availability

The data that support the findings of this study are available in the Supporting Information of this article.

## References

[1] M. Glikson , J. C. Nielsen , M. B. Kronborg , et al., “ESC Guidelines on Cardiac Pacing and CRT,” European Heart Journal 42, no. 35 (2021): 3427–3520. 10.1093/eurheartj/ehab364 34455430

[2] P. A. Heidenreich , B. Bozkurt , D. Aguilar , et al., “AHA/ACC/HFSA Guideline for Heart Failure Management,” Journal of the American College of Cardiology 79, no. 17 (2022): e263–e421. 10.1016/j.jacc.2021.12.012 35379503

[3] C. Daubert , N. Behar , R. P. Martins , et al., “Avoiding Non‐Responders to CRT,” European Heart Journal 38, no. 19 (2017): 1463–1472. 10.1093/eurheartj/ehw270 27371720

[4] M. K. Chung , K. K. Patton , C. P. Lau , et al., “HRS/APHRS/LAHRS Guideline on Cardiac Physiologic Pacing,” Journal of Arrhythmia 39, no. 5 (2023): 681–756. 10.1002/joa3.12872 PMC10549836 37799799

[5] P. Vijayaraman , M. G. Chelu , K. Curila , et al., “Cardiac Conduction System Pacing: A Comprehensive Update,” JACC: Clinical Electrophysiology 9, no. 11 (2023): 2358–2387. 10.1016/j.jacep.2023.06.005 37589646

[6] Y. Wang , H. Zhu , X. Hou , et al., “Randomized Trial of Left Bundle Branch Vs Biventricular Pacing for CRT,” Journal of the American College of Cardiology 80, no. 13 (2022): 1205–1216. 10.1016/j.jacc.2022.07.019 36137670

[7] D. Žižek , T. Žlahtič , M. Mrak , et al., “CSP Vs BVP for CRT: The CSP‐SYNC Study,” Europace 27, no. 9 (2025): euaf192. 10.1093/europace/euaf192 PMC13223735 40848259

[8] X. Chen , X. Liu , R. Li , et al., “HeartSync‐LBBP Trial,” JAMA Cardiology 11, no. 4 (2026): 352–359. 10.1001/jamacardio.2026.0083 PMC12980356 41811342

[9] A. Zimerman , A. dal Forno , L. E. Rohde , et al., “PhysioSync‐HF Trial,” JAMA Cardiology 11, no. 4 (2026): 360–368. 10.1001/jamacardio.2026.0101 PMC12980360 41811324

[10] F. Felix, I , M. Collini , R. Fonseca , et al., “CSP Vs BiVP in CRT: Systematic Review and Meta‐Analysis,” Heart Rhythm 21, no. 6 (2024): 881–889. 10.1016/j.hrthm.2024.02.035 38382686

[11] M. Pujol‐López , F. R. Graterol , R. Borràs , et al., “CONSYST‐CRT Trial,” JACC: Clinical Electrophysiology 11, no. 8 (2025): 1820–1831. 10.1016/j.jacep.2025.03.024 40372330

[12] M. Pujol‐Lopez , R. Jiménez‐Arjona , P. Garre , et al., “LEVEL‐AT Trial,” JACC: Clinical Electrophysiology 8, no. 11 (2022): 1431–1445. 10.1016/j.jacep.2022.08.001 36424012

[13] G. A. Upadhyay , P. Vijayaraman , H. M. Nayak , et al., “His Corrective Pacing or BVP for CRT,” Journal of the American College of Cardiology 74, no. 1 (2019): 157–159. 10.1016/j.jacc.2019.04.026 31078637

[14] J. Hartung and G. Knapp , “A Refined Method for Meta‐Analysis of Controlled Clinical Trials,” Statistics in Medicine 20, no. 24 (2001): 3875–3889. 10.1002/sim.1009 11782040

[15] J. IntHout , J. P. Ioannidis , M. M. Rovers , and J. J. Goeman , “Plea for Routinely Presenting Prediction Intervals,” BMJ Open 6, no. 7 (2016): e010247. 10.1136/bmjopen-2015-010247 PMC4947751 27406637

[16] J. A. C. Sterne , A. J. Sutton , J. P. A. Ioannidis , et al., “Recommendations for Funnel Plot Examination,” BMJ 343 (2011): d4002. 10.1136/bmj.d4002 21784880

[17] M. Vinther , N. Risum , J. H. Svendsen , et al., “His‐Alternative Trial,” JACC: Clinical Electrophysiology 7, no. 11 (2021): 1422–1432. 10.1016/j.jacep.2021.04.003 34167929

[18] E. A. Frandsen , J. Krøll , U. Winsløw , et al., “His Pacing Vs Biventricular Pacing for Cardiac Resynchronization Therapy: Long‐Term Follow‐Up From the His‐Alternative I Trial,” JACC Clin Electrophysiol 12 (2026): 1979–1991, 10.1016/j.jacep.2026.02.016. 41973899

[19] P. Vijayaraman , P. Pokharel , F. A. Subzposh , et al., “HOT‐CRT Trial,” JACC: Clinical Electrophysiology 9, no. 12 (2023): 2628–2638. 10.1016/j.jacep.2023.08.003 37715742

[20] D. L. Lustgarten , E. M. Crespo , I. Arkhipova‐Jenkins , et al., “His‐Bundle Pacing Vs BVP: Crossover Comparison,” Heart Rhythm 12, no. 7 (2015): 1548–1557. 10.1016/j.hrthm.2015.03.048 25828601

[21] W. Huang , S. Wang , L. Su , et al., “ALTERNATIVE‐AF Trial,” Heart Rhythm 19, no. 12 (2022): 1948–1955. 10.1016/j.hrthm.2022.07.009 35843465

[22] F. Kerkouri , L. Palud , J. B. Moreau , et al., “Left Bundle Branch Area Pacing Vs Biventricular Pacing for Cardiac Resynchronization: Propensity Score Analysis,” Journal of Arrhythmia 42, no. 3 (2026): e70359. 10.1002/joa3.70359 PMC13149756 42109833

