# Supplementary material for: Conduction System Pacing Versus Biventricular Pacing for Cardiac Resynchronization Therapy: A Systematic Review and Meta‐Analysis of Randomized Controlled Trials
Source: J Arrhythm. 2026 Oct 21;42(5):e70500. doi: 10.1002/joa3.70500 (PMC13685677; doi:10.1002/joa3.70500)
Supplement: Supplementary file 1 [file JOA3-42-e70500-s001.docx]

**Supplementary Appendix**

Systematic Review and Meta-Analysis of Conduction System Pacing Versus Biventricular Pacing for Cardiac Resynchronization Therapy

# **SUPPLEMENTARY CONTENTS**

Table S1. Search Strategy

Table S2. Detailed Study Characteristics

Table S3. Data Extraction — Continuous Outcomes

Table S4. Data Extraction — Binary Outcomes

Table S5. Risk of Bias Assessment (RoB 2) — Justification

Table S6. PRISMA 2020 Checklist

Figure S1. PRISMA 2020 Flow Diagram

Figure S2. Risk of Bias Traffic Light Plot

Figure S3. Risk of Bias Summary Bar Chart

Table S7. Leave-One-Out Sensitivity Analysis for ΔLVEF

Table S8. Exploratory Sensitivity and Subgroup Analyses

Table S9. CSP Definition and Capture-Confirmation by Trial

Table S10. Comparison with Prior Meta-Analyses

Table S11. GRADE Certainty of Evidence

Figure S4. Forest plot of pacing threshold (V).

# **Table S1. Search Strategy**

**Databases:** PubMed/MEDLINE, Embase (Ovid), Cochrane CENTRAL

**Date range:** July 2023 (end of the Felix et al. search) through 15 May 2026. This updated review builds on Felix et al. (Heart Rhythm 2024); randomized trials identified therein were carried forward and re-screened against our eligibility criteria (two crossover-design trials were moved to sensitivity analyses; the LEVEL-AT cohort was replaced by its extended-follow-up report, CONSYST-CRT). The updated search covered PubMed, Embase (Ovid), and Cochrane CENTRAL, supplemented by ClinicalTrials.gov and reference-list hand-searching. PROSPERO registration CRD420261407189 was registered on 27 May 2026, with the record subsequently amended (see the PROSPERO version history); the review was conducted between 30 April and 30 June 2026. The registered primary outcome was change in LVEF; paced QRS duration and the remaining measures were secondary outcomes. The analysis plan was amended during the review from the originally registered DerSimonian-Laird model (Mantel-Haenszel risk ratios for binary outcomes) to REML with the Knapp-Hartung adjustment across all outcomes, for more reliable small-sample inference; DerSimonian-Laird, Mantel-Haenszel, and Peto results are reported as sensitivity analyses. No date restriction was applied to the overall evidence base (inception through May 2026). Sensitivity analyses added after registration (standalone-CSP and high-crossover-exclusion) are labeled post hoc.

**Language:** No restrictions

**PubMed Search Strategy:**

#1: "conduction system pacing"[tiab] OR "His bundle pacing"[tiab] OR "His-bundle pacing"[tiab] OR "left bundle branch pacing"[tiab] OR "left bundle branch area pacing"[tiab] OR "LBBP"[tiab] OR "LBBAP"[tiab] OR "HBP"[tiab] OR "left bundle area pacing"[tiab]

#2: "biventricular pacing"[tiab] OR "cardiac resynchronization therapy"[tiab] OR "CRT"[tiab] OR "BiV"[tiab] OR "biventricular"[tiab]

#3: "randomized"[tiab] OR "randomised"[tiab] OR "RCT"[tiab] OR "randomized controlled trial"[pt] OR "controlled clinical trial"[pt]

#4: #1 AND #2 AND #3

#5: Filter: Publication date July 1, 2023 to May 15, 2026

#6: #4 AND #5

Embase (Ovid) Search Strategy: 1. (conduction system pacing or His bundle pacing or His-bundle pacing or left bundle branch pacing or left bundle branch area pacing or LBBP or LBBAP or HBP).ti,ab. 2. (biventricular pacing or cardiac resynchronization or cardiac resynchronisation or CRT or biventricular).ti,ab. or exp cardiac resynchronization therapy/ 3. (randomized or randomised or RCT).ti,ab. or randomized controlled trial/ or controlled clinical trial/ 4. 1 and 2 and 3 5. limit 4 to human

CENTRAL: (conduction system pacing OR His bundle pacing OR left bundle branch pacing) AND (biventricular pacing OR cardiac resynchronization) in Title/Abstract/Keywords.

ClinicalTrials.gov: "conduction system pacing" AND "biventricular" OR "cardiac resynchronization", filtered for completed studies with results.

# **Table S2. Detailed Study Characteristics**

**Panel A: Design and Demographics**

| **Study** | **N** | **CSP Type** | **FU (mo)** | **Sites** | **Country** | **LBBB %** | **Etiology NICM %** | **Age (y)** | **Female %** | **Baseline LVEF %** | **Baseline QRS ms** |
| --- | --- | --- | --- | --- | --- | --- | --- | --- | --- | --- | --- |
| **Upadhyay 2019** | 40 | HBP | 12 | 4 | USA | 82 | NR | 65 | 37 | 27 | 170 |
| **Vinther 2021** | 50 | HBP | 6 | 1 | Denmark | 100 | 78 | 66 | 36 | 29 | 172 |
| **Wang 2022** | 40 | LBBAP | 6 | 1 | China | 100 | 100 | 64 | 50 | 29 | 172 |
| **Vijayaraman 2023** | 100 | HOT-CRT | 6 | 1 | USA | 71 | 61 | 70 | 31 | 28 | 155 |
| **Pujol-López 2025** | 134 | LBBAP | 12 | 1 | Spain | 56 | 66 | 69 | 27 | 28 | 175 |
| **Žižek 2025** | 62 | LBBAP | 6 | 1 | Slovenia | 100 | 68 | 68 | 30 | 29 | 174 |
| **Chen 2026** | 200 | LBBP | 36 | 6 | China | 100 | 82 | 65 | 32 | 28 | 168 |
| **Zimerman 2026** | 173 | Mixed* | 12 | 14 | Brazil | 95 | 87 | 62 | 50 | 26 | 180 |

**Mixed: 69% LBBAP, 20% deep septal, 9% LOT-CRT, 2.5% His. NR = not reported. FU = follow-up. NICM = non-ischemic cardiomyopathy. CONSYST-CRT replaces LEVEL-AT (same cohort, extended follow-up). CSP-strategy classification and left bundle capture-confirmation status for each trial are detailed in Table S9; Vijayaraman 2023 (HOT-CRT) is a hybrid strategy incorporating an adjunctive coronary-sinus/left ventricular lead.*

# **Table S3. Data Extraction — Continuous Outcomes**

**Panel A: ΔLVEF (% change from baseline)**

| **Study** | **n CSP** | **CSP mean±SD** | **n BVP** | **BVP mean±SD** | **Note** |
| --- | --- | --- | --- | --- | --- |
| **Upadhyay 2019** | 16 | 6.9±11.5 | 24 | 4.5±7.2 | As-treated |
| **Vinther 2021** | 25 | 16.0±9.0 | 25 | 13.0±7.0 | ITT |
| **Wang 2022** | 20 | 19.4±13.2 | 20 | 16.5±9.4 | ITT |
| **Vijayaraman 2023** | 50 | 12.4±10.1 | 50 | 8.0±10.1 | ITT |
| **Pujol-López 2025** | 67 | 14.1±10.5 | 67 | 14.4±9.9 | ITT, 12mo |
| **Žižek 2025** | 31 | 14.0±7.7 | 31 | 8.5±7.7 | SD from CI |
| **Chen 2026** | 100 | 16.7±9.6 | 100 | 11.1±7.4 | 6mo data |
| **Zimerman 2026** | 87 | 8.0±11.0 | 86 | 12.0±11.0 | ITT, 12mo |

*SD = standard deviation. CI = confidence interval. CSP-SYNC SDs imputed from 95% CIs: SE = (upper−lower)/3.92, SD = SE × √n. HeartSync-LBBP uses 6-month LVEF for consistency; 36-month data used in sensitivity. PhysioSync-HF ΔLVEF calculated from Table 2 (change = post − baseline). His-SYNC (Upadhyay 2019) arm sizes (16 CSP, 24 BVP) reflect as-treated allocation after 48% crossover, as reported in the primary publication; the trial's intention-to-treat analysis retained the original randomized allocation. A sensitivity analysis excluding high-crossover trials (His-SYNC, CONSYST-CRT) is reported in Table S8.*

**Panel B: Paced QRS Duration (ms, absolute post-implant)**

| **Study** | **n CSP** | **CSP mean±SD** | **n BVP** | **BVP mean±SD** | **Note** |
| --- | --- | --- | --- | --- | --- |
| **Upadhyay 2019** | 16 | 125.0±22.0 | 24 | 164.0±25.0 |  |
| **Vinther 2021** | 25 | 134.0±15.0 | 25 | 131.0±20.0 |  |
| **Wang 2022** | 20 | 131.5±12.5 | 20 | 136.6±12.9 |  |
| **Vijayaraman 2023** | 50 | 137.0±20.0 | 50 | 141.0±19.0 |  |
| **Pujol-López 2025** | 67 | 126.0±17.0 | 67 | 131.0±13.0 |  |
| **Žižek 2025** | 31 | 143.0±19.0 | 31 | 138.0±19.0 |  |
| **Chen 2026** | 100 | 120.6±18.1 | 100 | 137.4±15.8 |  |
| **Zimerman 2026** | 87 | 122.0±25.0 | 86 | 126.0±21.0 |  |

# **Table S4. Data Extraction — Binary Outcomes**

| **Study** | **HFH CSP** | **HFH BVP** | **Death CSP** | **Death BVP** | **Comp CSP** | **Comp BVP** | **n CSP** | **n BVP** |
| --- | --- | --- | --- | --- | --- | --- | --- | --- |
| **Upadhyay 2019** | NR | NR | NR | NR | 1 | 3 | 16 | 24 |
| **Vinther 2021** | 0 | 0 | 0 | 0 | 1 | 1 | 25 | 25 |
| **Wang 2022** | 0 | 0 | 0 | 0 | 1 | 1 | 20 | 20 |
| **Vijayaraman 2023** | 7 | 2 | 2 | 2 | 3 | 10 | 50 | 50 |
| **Pujol-López 2025** | 5 | 8 | 3 | 4 | 8 | 6 | 67 | 67 |
| **Žižek 2025** | 2 | 7 | 2 | 1 | 2 | 2 | 31 | 31 |
| **Chen 2026** | 7 | 28 | 2 | 5 | 0 | 3 | 100 | 100 |
| **Zimerman 2026** | 6 | 4 | 11 | 4 | 10 | 7 | 87 | 86 |

*HFH = heart failure hospitalization; Comp = periprocedural/device-related complications; NR = not reported. Upadhyay 2019 did not separately report HFH or mortality counts. CONSYST-CRT complications include late adverse events requiring reintervention. 0.5 continuity correction applied to zero-cell studies in meta-analysis.*

# **Table S5. Risk of Bias Assessment — Justification**

| **Study** | **D1** | **D2** | **D3** | **D4** | **D5** | **Overall** | **Justification** |
| --- | --- | --- | --- | --- | --- | --- | --- |
| **Upadhyay 2019** | Low | Some concerns | Low | Some concerns | Low | Some concerns | Adequate randomization. High crossover (48% CSP→BVP). Echocardiographer blinding unclear. Small sample (n=40). |
| **Vinther 2021** | Low | Some concerns | Low | Low | Low | Some concerns | Adequate randomization. 12% crossover CSP→BVP. Echocardiographers blinded. Single-center. |
| **Wang 2022** | Low | Low | Low | Low | Low | Low | Block randomization. No crossover. Echocardiographers blinded. LBBB-only, single-center. |
| **Vijayaraman 2023** | Low | Some concerns | Low | Some concerns | Low | Some concerns | Adequate randomization. Hybrid HOT-CRT (His/LBBAP + LV lead). Unblinded design. Single-center. |
| **Pujol-López 2025** | Low | Some concerns | Low | Low | Low | Some concerns | Block randomization. 26.9% crossover CSP→BVP (mostly IVCD). Blinded echocardiographers. Single-center. Replaces LEVEL-AT. |
| **Žižek 2025** | Low | Low | Low | Low | Low | Low | Stratified randomization. 3.2% crossover. Blinded echocardiographers. Strict Strauss LBBB. Single-center. |
| **Chen 2026** | Low | Low | Low | Low | Low | Low | 1:1 randomization. 2% crossover. Blinded echocardiographers. Independent endpoint adjudication. Multicenter (6 sites). Strict LBBP criteria. |
| **Zimerman 2026** | Low | Some concerns | Low | Low | Low | Some concerns | 1:1 randomization. 6.9% crossover CSP→BVP. Core lab blinded for echo and ECG. Independent events committee. Multicenter (14 sites). Variable operator CSP experience. Stylet-driven leads only. |

*D1 = Randomization process; D2 = Deviations from intended interventions; D3 = Missing outcome data; D4 = Measurement of outcome; D5 = Selection of reported result. Assessed using Cochrane Risk of Bias 2 (RoB 2) tool.*

# **Table S6. PRISMA 2020 Checklist**

Complete PRISMA 2020 checklist. All 27 items reported; “Location” indicates where each item appears in the manuscript or Supplementary Appendix.

| **Item** | **PRISMA 2020 checklist item** | **Location** |
| --- | --- | --- |
| 1 | Title – identify as systematic review | Title page |
| 2 | Abstract (structured summary) | Abstract |
| 3 | Rationale | Introduction |
| 4 | Objectives / question | Introduction (final paragraph) |
| 5 | Eligibility criteria | Methods – Search Strategy and Study Selection |
| 6 | Information sources | Methods; Table S1 |
| 7 | Search strategy (full) | Table S1 (PubMed, Embase, CENTRAL) |
| 8 | Selection process | Methods; Figure S1 |
| 9 | Data collection process | Methods – Data Extraction |
| 10a | Data items – outcomes | Methods – Outcomes; Tables S3–S4 |
| 10b | Data items – other variables | Table S2 |
| 11 | Study risk of bias assessment | Methods; Table S5; Figs S2–S3 |
| 12 | Effect measures | Methods – Statistical Analysis |
| 13a–13f | Synthesis methods | Methods – Statistical Analysis |
| 14 | Reporting bias assessment | Methods (not undertaken; funnel plots and formal tests unreliable, k<10) |
| 15 | Certainty assessment (GRADE) | Methods; Table S11 |
| 16a | Study selection results | Results; Fig S1 (246+8 records; 187 screened; 164 excluded; 23 full-text; 19 excluded w/ reasons; 8 included) |
| 16b | Excluded studies with reasons | Figure S1 |
| 17 | Study characteristics | Table 1; Table S2 |
| 18 | Risk of bias in studies | Figs S2–S3; Table S5 |
| 19 | Results of individual studies | Tables S3–S4; forest plots |
| 20a–20d | Results of syntheses | Results; Table 2; Figs 1–7; Tables S7–S8 |
| 21 | Reporting biases | Discussion – Limitations |
| 22 | Certainty of evidence | Discussion; Table S11 |
| 23a–23d | Discussion | Discussion |
| 24a | Registration & protocol | PROSPERO CRD420261407189; Table S1 |
| 24b | Registration number | CRD420261407189 |
| 24c | Protocol amendments | Table S1 (estimator amended: registered DL/MH to REML+KH per PROSPERO version history; post hoc analyses disclosed) |
| 25 | Support / funding | Title page (none) |
| 26 | Competing interests | Title page (none declared) |
| 27 | Availability of data/code | Available from corresponding author; R code in Supplement |

# **Figure S1. PRISMA 2020 Flow Diagram**

# **Figure S2. Risk of Bias Traffic Light Plot**

# **Figure S3. Risk of Bias Summary Bar Chart**

# **Table S7. Leave-One-Out Sensitivity Analysis — ΔLVEF**

Each row shows the pooled estimate (REML + Knapp-Hartung) when the named study is excluded.

| **Study Excluded** | **k** | **MD** | **95% CI** | **P** | **I²** |
| --- | --- | --- | --- | --- | --- |
| None (all 8) | 8 | 2.40 | (−0.49, 5.29) | 0.091 | 74.5% |
| Upadhyay 2019 | 7 | 2.40 | (−0.98, 5.78) | 0.133 | 78.1% |
| Vinther 2021 | 7 | 2.32 | (−1.10, 5.74) | 0.148 | 78.1% |
| Wang 2022 | 7 | 2.36 | (−1.00, 5.71) | 0.136 | 78.1% |
| Vijayaraman 2023 | 7 | 2.10 | (−1.25, 5.45) | 0.176 | 77.4% |
| Pujol-López 2025 | 7 | 2.84 | (−0.43, 6.12) | 0.078 | 75.3% |
| Žižek 2025 | 7 | 1.92 | (−1.29, 5.14) | 0.194 | 76.0% |
| Chen 2026 | 7 | 1.77 | (−1.42, 4.96) | 0.224 | 67.9% |
| Zimerman 2026 | 7 | 3.61 | (1.49, 5.73) | 0.006 | 32.0% |

*Excluding PhysioSync-HF (Zimerman 2026) restores statistical significance and reduces I² from 74.5% to 32.0%, confirming it as the dominant source of heterogeneity. No other single study exclusion changes the direction or significance of the pooled estimate.*

**Table S8. Exploratory Sensitivity and Subgroup Analyses**

**Panel A. ΔLVEF subgroup and sensitivity analyses (REML + Knapp-Hartung). Positive MD favors CSP.**

| **Analysis** | **k** | **N** | **MD (95% CI)** | **P** | **I²** |
| --- | --- | --- | --- | --- | --- |
| Primary (all trials) | 8 | 799 | 2.40 (−0.49, 5.29) | 0.091 | 74.5% |
| Exclude PhysioSync-HF | 7 | 626 | 3.61 (1.49, 5.73) | 0.006 | 32% |
| Exclusively LBBB (any CSP) | 4 | 352 | 5.00 (3.01, 6.98) | 0.004 | 0% |
| Confirmed LBBAP/LBBP in LBBB | 3 | 302 | 5.37 (3.23, 7.52) | 0.008 | 0% |
| Standalone CSP (excl HOT-CRT + mixed) | 6 | 526 | 3.43 (0.83, 6.02) | 0.019 | 43% |
| LBBAP/LBBP trials | 4 | 436 | 3.59 (−1.11, 8.28) | 0.093 | 64% |
| HBP-only | 2 | 90 | 2.80 (−0.79, 6.39) | 0.064 | 0% |
| Exclude high-crossover (≥25%) | 6 | 625 | 2.89 (−1.07, 6.85) | 0.119 | 79% |
| Longest available follow-up (HeartSync-LBBP 36-mo) | 8 | 799 | 2.38 (−0.51, 5.28) | 0.093 | 73% |
| DerSimonian–Laird (all trials) | 8 | 799 | 2.40 (−0.36, 5.16) | 0.088 | — |

**Panel B. Binary-outcome method sensitivity for sparse data. Primary model is the random-effects risk ratio (RR); Peto OR and Mantel-Haenszel (MH) risk difference are shown as sensitivity analyses.**

| **Outcome** | **Primary RR (95% CI)** | **Peto OR (95% CI)** | **MH risk difference (95% CI)** |
| --- | --- | --- | --- |
| HF hospitalization | 0.69 (0.18, 2.67) | 0.51 (0.32, 0.83) | −0.053 (−0.211, 0.105) |
| All-cause mortality | 1.17 (0.42, 3.23) | 1.26 (0.64, 2.46) | +0.005 (−0.049, 0.059) |
| Complications | 0.84 (0.45, 1.54) | 0.79 (0.46, 1.35) | −0.020 (−0.053, 0.013) |

*All analyses are exploratory and hypothesis-generating. The Peto OR for HF hospitalization is nominally significant, but the Peto method is biased when arm sizes and event counts are imbalanced (here driven by HeartSync-LBBP: 7 vs 28 events); the underpowered, non-significant primary risk ratio remains the basis for inference. Longest-available-follow-up sensitivity: HeartSync-LBBP (Chen 2026) is the only included trial with follow-up beyond 12 months. Substituting its 36-month ΔLVEF (LBBP 47.3% vs BVP 41.5%; MD +5.6%) for the 6-month value yields a pooled MD of +2.38% (95% CI −0.51 to 5.28; P=0.093), essentially unchanged from the primary estimate.*

**Table S9. CSP Definition and Left Bundle Capture-Confirmation by Trial**

| **Trial** | **CSP strategy** | **LBB capture confirmed?** | **Adjunctive LV lead / hybrid** | **Classification** |
| --- | --- | --- | --- | --- |
| His-SYNC (Upadhyay 2019) | His-bundle pacing (His-corrective) | His capture targeted per criteria | No | HBP |
| His-Alternative (Vinther 2021) | His-bundle pacing | His capture confirmed | No | HBP |
| Wang 2022 | LBBAP | LBB-area capture criteria applied | No | LBBAP |
| HOT-CRT (Vijayaraman 2023) | His/LBBAP + coronary-sinus LV lead | Variable | Yes (adjunctive LV lead) | Hybrid (HOT-CRT) |
| CONSYST-CRT (Pujol-López 2025) | LBBAP (may include LV septal) | Not uniformly confirmed | No | LBBAP |
| CSP-SYNC (Žižek 2025) | LBBAP; strict Strauss LBBB entry | LBB capture per criteria | No | LBBAP |
| HeartSync-LBBP (Chen 2026) | LBBP | ECG-confirmed LBB capture (strict) | No | LBBP (strict) |
| PhysioSync-HF (Zimerman 2026) | Mixed: 69% LBBAP, 20% deep septal, 9% LOT-CRT, 2.5% His | Partial (deep-septal subset unconfirmed) | LOT-CRT subset | Mixed |

*The pooled CSP arm is clinically heterogeneous. Sensitivity analyses separating standalone CSP from hybrid strategies (HOT-CRT) and mixed/deep-septal pacing are reported in Table S8.*

**Table S10. Comparison with Prior Meta-Analyses**

| **Feature** | **Felix et al. 2024 (Heart Rhythm)** | **Present review** |
| --- | --- | --- |
| Study designs included | RCTs + observational cohorts | Parallel-group RCTs only |
| Search end date | ~mid-2023 | May 2026 (updated search) |
| Number of RCTs | Fewer; predominantly pre-2023 | 8 RCTs, incl. 2025–26 trials (HeartSync-LBBP, PhysioSync-HF, CSP-SYNC, CONSYST-CRT) |
| CSP definition handling | Pooled | Pooled + subtype/hybrid sensitivity (Table S9) |
| Estimator | DerSimonian–Laird | REML + Knapp–Hartung; prediction intervals |
| Certainty of evidence | Not graded | GRADE (Table S11) |
| Sparse-data handling | Standard | Peto OR + MH risk difference sensitivity (Table S8) |
| Confirmed-CSP-in-LBBB analysis | Not isolated | Reported (Table S8) |

*This review's incremental contribution: restriction to randomized evidence; inclusion of the newest divergent RCTs (including the first trial reporting CSP inferiority, PhysioSync-HF); small-sample-appropriate estimation with prediction intervals; explicit handling of CSP definitional heterogeneity; GRADE certainty rating; and sparse-data sensitivity analyses. Other physiologic-pacing meta-analyses have been predominantly observational or mixed and are discussed in the main text.*

**Table S11. GRADE Certainty of Evidence**

| **Outcome** | **Trials (N)** | **Effect (95% CI)** | **Certainty** | **Reasons for downgrading** |
| --- | --- | --- | --- | --- |
| ΔLVEF | 8 (799) | MD +2.40% (−0.49, 5.29) | Low | Inconsistency (I²=75%); imprecision (CI crosses null) |
| Paced QRS | 8 (799) | MD −7.6 ms (−18.4, 3.3) | Low | Inconsistency (I²=85%); imprecision |
| NYHA class | 5 (597) | SMD −0.33 (−0.80, 0.14) | Low | Inconsistency; imprecision |
| HF hospitalization | 5 (669) | RR 0.69 (0.18, 2.67) | Very low | Very serious imprecision (few events); inconsistency |
| All-cause mortality | 5 (669) | RR 1.17 (0.42, 3.23) | Very low | Very serious imprecision (36 events total) |
| Complications | 8 (799) | RR 0.84 (0.45, 1.54) | Low | Imprecision (sparse events) |

*All outcomes began as high certainty (randomized evidence) and were downgraded per GRADE. Risk of bias (“some concerns” in 5 of 8 trials) further limits certainty for several outcomes. No outcome met criteria for high or moderate certainty.*

**Figure S4. Forest plot of pacing threshold (V).**
